# An unusual cause of food-induced anaphylaxis in mothers

**DOI:** 10.1186/s40413-016-0136-x

**Published:** 2017-02-10

**Authors:** J. Y. Soh, W. C. Chiang, C. H. Huang, C. K. Woo, I. Ibrahim, K. Heng, A. Pramanick, B. W. Lee

**Affiliations:** 1 Department of Paediatrics, National University Hospital, Tower Block Level 12, 1E Kent Ridge Road, Singapore, 119228 Singapore; 2 Department of Paediatrics, Yong Loo Lin School of Medicine, National University of Singapore, Singapore, Singapore; 3 Department of Paediatrics, Kandang Kerbau Women’s and Children’s Hospital, Singapore, Singapore; 4 Department of Medicine, Gleneagles Hospital Kuala Lumpur, Kuala Lumpur, Malaysia; 5 Emergency Medicine Department, National University Hospital, National University Health System, Singapore, Singapore; 6 Department of Surgery, Yong Loo Lin School of Medicine, National University of Singapore, Singapore, Singapore; 7 Emergency Department, Tan Tock Seng Hospital, Singapore, Singapore; 8 Department of Obstetrics and Gynaecology, Kandang Kerbau Women’s and Children’s Hospital, Singapore, Singapore

**Keywords:** Anaphylaxis, Pregnancy, Galacto-Oligosaccharide

## Abstract

**Background:**

Galacto-oligosaccharides (GOS) are prebiotics added to commercial milk formula of infants and mothers. In recent years, cases of allergy related to GOS in atopic children have been reported in the South East Asian region.

**Case presentations:**

We describe a series of pregnant (*n* = 4) and lactating mothers (*n* = 2) who developed anaphylactic reactions after consumption of maternal milk formula containing GOS. All six subjects had pre-existing atopy and a positive skin prick test to GOS and 5/5 of the subjects who were tested had positive basophil activation tests to GOS. All of the mothers and their babies had normal neonatal outcomes after the reactions.

**Conclusions:**

The supplementation of GOS into milk and beverages in the Asian region should take into account the rare chance of allergenicity of GOS in the atopic population.

## Background

Anaphylaxis in pregnancy is uncommon, and is often caused by antibiotics or drugs administered during labor [1–3]. It can result in maternal morbidity and neonatal death [1, 4].

Galacto-oligosaccharides (GOS) are a mixture of sugar chains up to seven units in length, which are synthesized by β galactosidases which transfer galactose to lactose [5]. β galactosidase enzymes from different sources (e.g. fungal, bacterial) are known and used for GOS production [5, 6]. The GOS produced by enzymes from different sources result in GOS of varying composition. GOS function as prebiotics in infants [7], with promising results in mouse studies suggesting effects on immune markers associated with tolerance, diminished allergic sensitization in female offspring, and reduced allergic asthma symptoms in male offspring [8–10]. The supplementation of the prebiotic mixture GOS/FOS (fructo-oligosaccharides) in the last trimester of pregnancy was investigated in a clinical trial. The prebiotic mixture was well tolerated by the mothers and no side-effects were observed in mothers or their offspring. Furthermore, the prebiotics also altered the maternal gut composition towards the bacteria thought to be favorable towards immune tolerance, although this effect was not shown to be transferable to the newborn infant [11].

GOS does not occur naturally in foods, although a number of GOS-structures have been found in high concentrations in human colostrum [12]. It is a manufactured prebiotic with a safe history of use and US FDA-approved GRAS (Generally Recognized As Safe) status [13, 14]. GOS is mainly applied as ingredient in infant milk formulae worldwide (Asia, Australia, America, Europe), but has also been supplemented to food products in recent years (e.g. yoghurt, drinks, bars, food supplements). When present, GOS is an added ingredient to milk formulae at up to 8 g per liter.

Unfortunately, application of GOS in milk formulae and food products was accompanied by reports of rare anaphylactic/allergic reactions to GOS in the Asian region: Japan, Singapore, Malaysia, Vietnam and Thailand [15–17]. The severity of the reactions varies and affected both adults as well as children as young as preschool age. Some developed anaphylaxis and required hospitalization. This is unusual as there is evidence to suggest an IgE-mediated reaction [16], yet GOS is a carbohydrate rather than a protein. GOS is a small molecule and thus is unlikely to crosslink IgE on the surface of mast cells. However, it may be immunogenic when coupled to carrier proteins, as demonstrated in guinea pigs [15]. Protein contamination from the manufacturing process for GOS has been ruled out as the mixtures have been shown to have undetectable to low protein levels (50 ng protein per 1 mg of GOS) [16] and our earlier study [16] also demonstrated the absence of IgE sensitization (skin prick tests and basophil activation tests) to the enzyme β galactosidase.

We have previously described GOS allergy in older children and an adult male [16]. Since then, we have been performing further surveillance study of GOS allergy seen in the Accident and Emergency Departments of three local hospitals. Based on this surveillance, new cases of GOS allergy were identified in pregnant and lactating mothers. Here, we report a series of mothers who presented with acute GOS allergy after consumption of GOS-containing milk formula.

## Case presentations

From November 2012 to May 2016, six mothers developed allergic reactions after consuming cow’s milk-based formulae supplemented with GOSproduced with β galactosidase from *Bacillus circulans.*. These women were either referred to our institution or were seen in the Accident and Emergency department of the National University Hospital of Singapore, which is one of the tertiary hospitals in the country. All subjects resided in Singapore except for one subject who was referred by her allergist from Kuala Lumpur, Malaysia. These women were aged 26–40 years old. All were previously ingesting dairy products without problems. Four had their allergic reactions to GOS containing formula during pregnancy, three in the third trimester and one in the first trimester; the other two had reactions in the postnatal period.

The suspected allergen was a commercial cow’s milk formulated for pre- and postnatal mothers. All reactions occurred at the first ingestion of the suspected milk formula, suggesting a cross-reaction typical of GOS allergy in our population [16]; the mothers had not taken these formulae previously. Reactions occurred 15–30 min after ingestion of the formula (Table 1). The amount ingested ranged from 100 ml to 250 ml (approximately 0.72 to 1.9gm of GOS). All had nasal symptoms; 4/6 had angioedema of the eyes (Table 1). Five of six women had anaphylaxis as per WAO criteria [13]. These criteria consist of any one of the following three:

**Table 1:** Clinical characteristics of mothers with acute allergic reactions to galacto-oligosaccharide (GOS) in mothers’ milk formula

Subject ID	Pregnancy status	History of atopy	Volume of milk consumed (ml)	Amount of GOS consumed (g)	Time to onset of symptoms (min)	Symptoms	Adrenaline received	Skin prick test to GOS wheal size (mm)
P1	Prenatal	Chronic rhinitis	Unknown	Unknown	30	Rhinorrhea, Throat tightness and itch, difficulty breathing, hives, red skin	Yes, dose unknown	8
P2	Prenatal	Chronic rhinitis, childhood asthma, sea urchin allergy, bird’s nest allergy	200	1.5	15	Eye swelling, congested nose, itchy throat, cough, wheezing	No	6
P3	Prenatal	Aspirin allergy	250	1.9	30	Eye and lip swelling, congested nose, itchy throat, hives, red skin	500 μg	6
P4	Postnatal	Chronic rhinitis, allergy to prawns and crabs, Bactrim allergy	200–250	1.5–1.9	30	Eye swelling, congested nose, throat tightness, cough, red skin	No	6
P5	Prenatal	Chronic rhinitis	100	0.72 g	40	Angioedema, running nose, watery eyes, sneezing,itchy throat, throat tightness, cough, wheezing, hoarseness of voice	No	5
P6	Postnatal	Chronic rhinitis	240	1.8	30	Eye swelling, facial itch, congested/running nose	No	4

Legend: *GOS* Galacto-oligosaccharide

1. acute-onset illness with involvement of the skin and/or mucosae, and at least one of respiratory compromise/reduced blood pressure/symptoms of end-organ dysfunction; or
2. rapid occurrence after exposure to a likely allergen for a patient, of at least two of skin-mucosae involvement/respiratory compromise/reduced blood pressure or symptoms thereof/persistent gastrointestinal symptoms; or
3. reduced blood pressure after exposure to a known allergen for the patient).

Four of the patients promptly visited the Emergency Department for their symptoms. Of the five women who had anaphylaxis, two received intramuscular adrenaline. None of the patients required hospitalization.

All six women were atopic as characterized by sensitisation to house dust mites in Singapore [18], and had positive skin prick tests to the house dust mites *Dermatophagoides pteronyssinus, Dermatophagoides farinae,* and *Blomia tropicalis.* The subjects did not have any other documented allergies. It is not uncommon that in our tropical environment atopics are monosensitised to dust mites only [18]. No other allergy tests other than to dust mites were performed, as our subjects reported respiratory allergies as their comorbid condition which is strongly associated with dust mite monosensitization in Singapore.

**Fig. 1:**
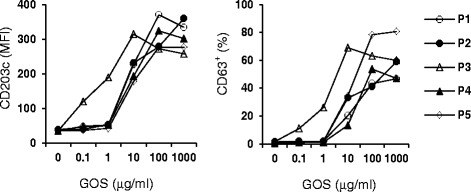
Dose-dependent effect of GOS on activation of basophils. The expression of CD203c (*left panel*) and CD63 (*right panel*) on basophils after stimulating with various concentration of GOS were analyzed by flow cytometry. P1-5 denotes patients 1–5 as described in Table. Basophil activation assay was performed as described previously [11]. The cells stimulated with anti-IgE Ab (1ug/ml) was used as the positive control (data not shown)

Five of six subjects had a history of respiratory allergies (chronic rhinitis, asthma). All patients had positive skin prick test to GOS, and all five women who were tested had positive basophil activation tests to GOS (Figure 1). The GOS used in this investigation was kindly provided by Friesland Campina (Amersfoort, Netherlands). The sixth patient was from Kuala Lumpur, Malaysia, hence the blood sampling for basophil activation test was not feasible.

The babies born to the four mothers who had reactions during pregnancy, were of normal birth weight with no congenital defects or perinatal problems. There have been no recurrences of the mothers’ allergic reactions after avoiding GOS-containing milks.

## Discussion

Anaphylaxis in pregnancy is rare, with one study estimating prevalence near or at the time of delivery as 2.7 cases of anaphylaxis per 100,000 deliveries [2]. In this report, we describe an unusual cause of anaphylaxis in mothers, namely to GOS which was added as a prebiotic supplement to milk formula. The new formulation of milk formula intended for pregnant and lactating women in Singapore, has had GOS removed from its ingredients. Therefore, it is unlikely that further anaphylactic reactions to GOS will occur in pregnant/lactating women. Of interest is the geographic restriction of cases of GOS-related allergy to South East Asia despite GOS being available worldwide, suggesting that the primary sensitizer is likewise specific to this region. It may be caused by an insect bite, akin to alpha-gal allergy associated with tick bites in the United States of America [19], Australia [20], Europe [21] and more recently, Japan [22]. Mosquito bites are frequent in Asia and the species varies with geographic region. The mechanism of sensitization remains unknown. Commins et al [23] summarised the historical and scientific evidence pointing to tick bites as the cause of development of alpha-gal allergy. Hapten sensitization, where the individual develops sensitization to the oligosaccharide when bound to a carrier protein, seems a plausible explanation but requires further investigation.

In pregnant mothers, management of any anaphylaxis is similar to that in non-pregnant patients in most respects. Adrenaline, administered promptly via intramuscular injection, is first-line therapy. The additional factor is the presence of the foetus: the pregnant patient should be placed semi-recumbent on her left side with the lower extremities elevated, to prevent positional hypotension resulting from compression of the inferior vena cava by the gravid uterus. In addition, monitoring of the fetal heart rate is recommended in women more than 24 weeks pregnant, with persistent fetal distress being an indication to consider emergency cesarean section [24].

In maternal anaphylaxis, the foetus tends to come off worst [1, 4, 25–27]. Various reports in the literature describe neonatal deaths and neurological damage, related to maternal anaphylaxis after the mothers received antibiotics in the hospital [25–27]. Berenguer et al [4] recently reported the demise of a neonate who was born premature with hypoxic-ischemic injury, likely due to maternal anaphylaxis after receiving amoxycillin in the community setting.

It is further unclear as to how much of this fetal morbidity and mortality may be related to administration of adrenaline. Adrenaline is a potent vasoconstrictor. It is thus possible that adrenaline, especially at high cumulative doses, may cause uterine vasoconstriction with consequent fetal hypoperfusion and the consequences thereof, including brain damage and death [28, 29]. Thus, it may be prudent to temper the use of adrenaline if there are no severe or life-threatening features of anaphylaxis such as airway compromise or hypotension. In our series, only two of the mothers received adrenaline and all mothers and neonates had an excellent outcome. There were no biphasic reactions; however, our case series is small. Given that this is a relatively new phenomenon, the frequency of biphasic reactions is unclear. Thus, it may be prudent to admit these mothers for observation in the hospital ward.

As mentioned, of the five cases of GOS-related anaphylaxis described in the current case report, only two received intramuscular adrenaline. All of the mothers and their babies had normal neonatal outcomes after the reactions.

## Conclusion

GOS-related allergy has manifested as anaphylaxis due to milk formula ingestion in a small number of prenatal and postnatal mothers with pre-existing atopy. Since the new formulation of milk formula intended for pregnant and lactating women in Singapore does not contain GOS, it is unlikely that such GOS-related allergy cases will occur in the future. The supplementation of GOS into milk and beverages in the South East Asian region should take into account the rare chance of allergenicity of GOS in the atopic population.

## Abbreviation

GOS: Galacto-oligosaccharides
